# Challenges and Considerations in Identifying the Origin of Peritoneal Carcinomatosis: A Case Report

**DOI:** 10.7759/cureus.37469

**Published:** 2023-04-12

**Authors:** Chih-kai Huang, Jin-Hwang Liu, Jiann-shang Chou, Chuan-hsun Chang

**Affiliations:** 1 Department of Breast Surgery, Cheng Hsin General Hospital, Taipei, TWN; 2 Department of Hemato-oncology, Cheng Hsin General Hospital, Taipei, TWN; 3 Department of Pathology, Cheng Hsin General Hospital, Taipei, TWN

**Keywords:** 18f-fdg pet-ct scan, peritoneal neoplasm, lobular, gata3, breast carcinoma

## Abstract

Invasive lobular cancer (ILC) of the breast is the second most common type of invasive breast cancer. Clinical determination of the growth pattern of ILC of the breast is difficult. Furthermore, the ILC of the breast has a unique metastatic pattern that involves gastrointestinal and peritoneal sites. Our patient was initially misdiagnosed with left ovarian cancer based on the findings of positron emission tomography and computed tomography. Herein, we report a case of ILC of the breast presenting as peritoneal carcinomatosis. The ESMO Clinical Practice Guidelines for cancers of unknown primary sites were used in the diagnosis of the carcinoma of unknown primary origin. Image-guided biopsy and immunohistochemical staining are also useful in the diagnosis of these cancer types.

## Introduction

Invasive lobular cancer (ILC) of the breast accounts for approximately 10% of all breast cancers and is the second most common type of invasive breast cancer worldwide [1]. ILC of the breast is commonly estrogen receptor (ER)- or progesterone receptor (PR)-positive and HER2/neu-negative [2]. It is difficult to diagnose initially because it has an ambiguously palpable lesion with ill-defined margins, a unique metastatic pattern that involves gastrointestinal and peritoneal sites, and challenging images that report higher false-negative rates than other invasive breast cancers [3-5]. The patient was first diagnosed with left ovarian cancer with abdominal carcinomatosis according to the positron emission tomography/computed tomography (PET-CT) report. We learned an important lesson that PET-CT had merits when it comes to ILC. Furthermore, ILC of the breast is more sensitive to endocrine therapy than chemotherapy with respect to the duration of disease-free survival [6,7].

## Case presentation

**Figure 1:**
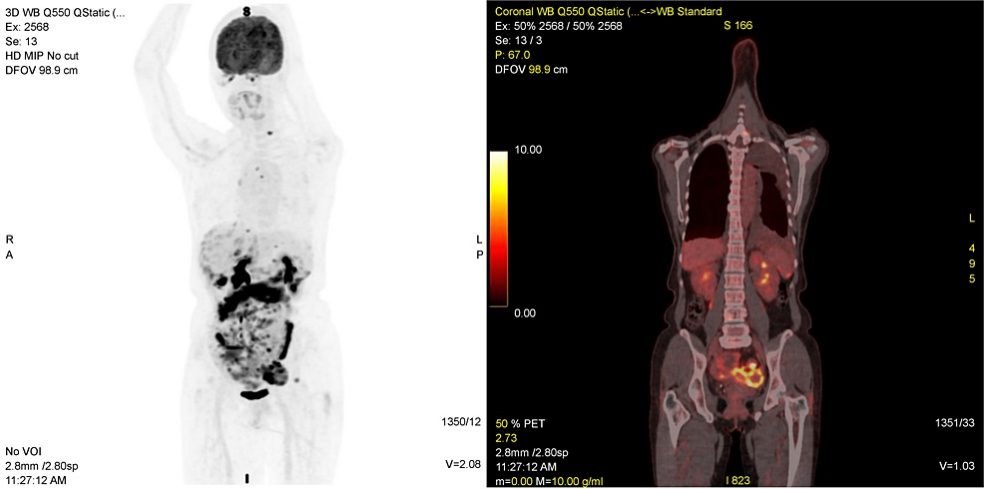
Disseminated FDG-avid foci in the peritoneum, mesentery, celiac LNs, paraaortic LNs, and omentum with a strong FDG-avid omentum cake in the mid-abdomen (SUVmax: 14.3), and cul-de-sac. The lower conspicuity foci were over the left breast. FDG: fluorodeoxyglucose; LN: lymph node

In December 2021, a 56-year-old woman was referred to the surgery department of Chen-Hsin Hospital with a history of a palpable 10 cm (largest diameter) mass in the pelvis that had grown over the previous six months. The chief concerns were unintentional weight loss of 10 kg and abdominal fullness. The patient had no personal or family history of cancer. She had left pelvic endometriosis and underwent conservative surgery with the excision of the endometriotic lesions 20 years earlier. No abnormal uterine bleeding was noted. Abdominal CT revealed peritoneal carcinomatosis, scanty bilateral pleural effusion, and ascites. No abnormalities were observed in the hemogram or serum biochemical profiles. Serum tumor markers revealed a CA-125 level of 217 U/mL (normal range: ≤ 35 U/mL) and a CA15-3 level of 57.7 U/mL (normal range: ≤ 25 U/mL). A PET-CT scan revealed 18F-fluorodeoxyglucose (FDG)-avid omentum implants in the mid-abdomen (SUVmax: 14.3) and solid FDG-avid foci in the left adnexa (3 cm, SUVmax: 13.3) (Figure 1). Upper and lower gastrointestinal endoscopic examinations revealed no abnormalities, and the presence of primary gastrointestinal cancer was unlikely. Sonography of the pelvis revealed ascites with a lobulated hypoechoic mass measuring 7.1 × 5.9 cm in the left adnexal region, suspected to be an ovarian or metastatic tumor. However, abnormal endometrial thickness was not noted.

Cytological examination of the pleural fluid showed malignant GATA3 (+) cells. Biopsies of the left adnexal mass and pelvic-peritoneal implants were performed. The tumor cells of the mesentery were immunoreactive for GATA-3 and nonreactive for ER and PR. The tumor cells in the left fallopian tube were GATA3 (+) and ER (+). The tumor cells in the mesocolon were GATA3 (+) and ER (-) (Table 1).

**Table 1:** Sites of biopsy and the immunohistochemistry (IHC) results. CT: computed tomography; ER: estrogen receptor; PR: progesterone receptor

Sites	Methods	IHC	Pathology
Breast, left, 4 o'clock position	Sono-guided biopsy	ER+, 60%, PR+, 60%, HER2/neu: Negative (score 0) MIB-1 labeling index: 10	Invasive lobular carcinoma
Mesointestine, large	Laparoscopic	GATA3(+), ER(-)	Metastatic carcinoma from breast
Fallopian tube, left	Laparoscopic	GATA3(+), ER(+)	Metastatic carcinoma from breast
Mesentery	CT-guided biopsy	GATA-3(+), ER (+)	Metastatic adenocarcinoma
Pleural fluid	Thoracocentesis	GATA3(+), ER(-), PAX-8(-), WT-1(-), CDX-2(-)	Malignancy

**Figure 2:**
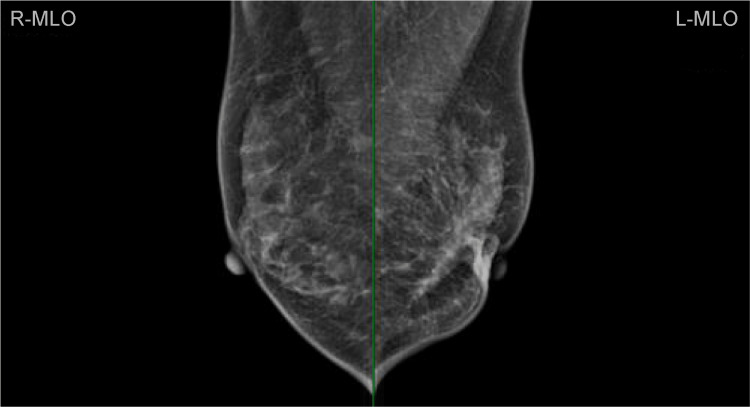
Architectural distortion is present in the left breast on the medial-lateral view.

Mammography revealed abnormal radiological features of relatively dense parenchyma over the left breast, with skin thickening and retraction in the outer region (Figure 2). A core biopsy of the breast mass was performed. A histological examination revealed an ILC located at the 4 o'clock position in the left breast. The immunohistochemical (IHC) results were positive for both ER (intermediate to strong, 60%) and PR (strong, 60%); however, there was a lack of HER-2/neu expression (score 0), whereas the MIB-1 labeling index was 10%.

Based on these findings, a diagnosis of stage IV ILC of the left breast with widespread systemic carcinomatosis was made.

## Discussion

Peritoneal carcinomatosis of unknown origin is difficult to diagnose and treat. Concomitant metastasis to the gastrointestinal, pelvic, and peritoneal regions is even more challenging [8] and the diagnostic strategy relies strongly on IHC analysis and the relevant tumor markers that are used to categorize the various tumor types [9]. Moreover, surgical exploration is required in some cases. The literature has also revealed that a minority of patients have occult appendiceal adenocarcinoma [10].

In females, diffuse peritoneal adenocarcinoma without an obvious primary tumor usually originates in the ovary or tissues with a similar histogenesis [11]. In our patient, the lesion was diagnosed as breast carcinoma. Patients with ovarian metastases from breast carcinoma have a worse prognosis compared with patients with independent breast and ovarian carcinomas [12]. Thus, distinguishing between independent primary and metastatic tumors is crucial. IHC staining for Pax-8, WT-1, and GATA3 was useful [6]. The negative PAX8 expression suggested a non-Müllerian origin [7], which showed the limited possibility of tumor metastasis from primary ovarian cancer. The diagnosis of breast carcinoma with diffuse peritoneal metastasis was thus established, which was confirmed by breast biopsy and IHC findings (Table 1).

Our patient had reported that endometriosis was surgically verified 20 years previously. Young women with endometriosis and many symptoms undergo more frequent surgeries and use additional therapies that may also contribute to the risk of breast cancer [13]. Although there may be little or no causative association between endometriosis and breast cancer, the relationship between them is likely a correlation because of their shared risk factors, including genetic risk factors (particularly BRCA mutations) [14].

Peritoneal carcinomatosis secondary to breast cancer has been reported to have a prevalence of 0.5%-0.7% [15]. Patients diagnosed with peritoneal carcinomatosis commonly present with high-grade ILC of the breast and advanced TNM stage [16]. ILC of the breast is often difficult to diagnose early because of a diffuse pattern of infiltration within the breast, resulting in a variety of subtle radiological appearances [17,18]. Notably, the ILC of the breast demonstrates lower conspicuity on 18F-FDG PET/CT [19]. In the present case, the radiologist first reported left ovarian cancer with abdominal carcinomatosis and revised the diagnosis after identifying the lesion in the left breast on 18F-FDG PET/CT.

In specific groups, such as peritoneal carcinomatosis of unknown origin, a survey of pelvic and gastrointestinal tumors should be performed first [20]. A basic workup should comprise a whole-body physical examination, basic blood and biochemical analyses, and serum assessment of tumor markers. Moreover, endoscopies should be problem-guided. 18F-FDG PET/CT should not be relied on for the detection of the primary tumor site [20]. Breast cancers such as ILC should be considered, especially in women with peritoneal carcinomatosis.

## Conclusions

In this article, we presented a case of ILC with peritoneal carcinomatosis, which was initially misdiagnosed based on PET/CT findings. The PET/CT findings were misleading because the ILC showed hypo-enhancement in 18F-FDG PET/CT. We consulted an oncologist and performed a general physical examination. A breast tumor was found, which was compatible with the IHC of the peritoneal biopsy. The affirmed origin of the peritoneal carcinomatosis determined the specific treatment regimen.

We should always consider breast cancer in patients with peritoneal metastasis. Image-guided biopsy in combination with IHC can often provide clues for the origin of the tumor.
